# EXPLORING AGE AND RACE DIFFERENCES IN THE REPORT OF ADVERSE CHILDHOOD EXPERIENCES AMONG WOMEN AGING IN CUSTODY

**DOI:** 10.1093/geroni/igac059.1908

**Published:** 2022-12-20

**Authors:** Alex Bishop, Claire Stolfa

**Affiliations:** Oklahoma State University, Stillwater, Oklahoma, United States; Oklahoma State University, Stillwater, Oklahoma, United States

## Abstract

Survey data for this study was collected from N = 544 women, aged 18 and older (M = 39.03; SD = 10.32), under correctional custody in Oklahoma. Participants reported an average of M = 4.94; SD = 2.94 childhood adversities. Chi-square analyses revealed that age was associated with the proportion of women who experienced parental divorce (χ(2, N = 525) = 8.03, p < .05., lived with a substance abuser (χ(1, N = 528) = 7.14, p < .05), lived with a mentally ill household member (χ(2, N = 524) = 15.04, p < .05) and lived with household member who went to prison (χ = (2, N = 528) = 18.99, p < .001). Race was also associated with the proportion of participants who reported experiencing a household member go to prison (χ(1, N = 538, = 15.92, p < .001). Furthermore, race was associated with the proportion of women who reported not feeling loved by family (χ(1, N = 525), = 4.87, p < .05), being physical hurt or injured by a parent (χ(2, 525) = 8.01 p < .05), and being verbally insulted or humiliated (χ = 7.38 (1, N = 537, p < .01). Results suggest that age and race are associated with the self-disclosure of past childhood adversities among women in custody. This has implications relative to how clinicians such as social workers, counselors, and psychiatrists provide rehabilitative services and programs to women-of-color aging under correctional control.

